# Interleukin-9 (IL-9) and NPM-ALK each generate mast cell hyperplasia as single ‘hit’ and cooperate in producing a mastocytosis-like disease in mice

**DOI:** 10.18632/oncotarget.115

**Published:** 2010-06-07

**Authors:** Hartmut Merz, Christian Kaehler, Kai P. Hoefig, Biggi Branke, Wolfgang Uckert, Roger Nadrowitz, Sabine-Cerny-Reiterer, Harald Herrmann, Alfred C. Feller, Peter Valent

**Affiliations:** ^1^Department of Pathology, Medical University of Schleswig-Holstein, Campus Luebeck, Luebeck, Germany; ^2^Institute for Molecular Immunology, Helmholtz Zentrum München, Germany; ^3^Max-Delbrueck-Center for Molecular Medicine, Berlin, Germany; ^4^Institute for Radiotherapy, Medical University of Schleswig-Holstein, Campus Lübeck, Luebeck, Germany; ^5^Department of Medicine I, Division of Hematology, Medical University of Vienna, Austria; ^6^Ludwig Boltzmann Cluster Oncology, Vienna, Austria

**Keywords:** IL-9, ALK, NPM, mast cells, mastocytosis, KIT

## Abstract

Mast cell neoplasms are characterized by abnormal growth and focal accumulation of mast cells (MC) in one or more organs. Although several cytokines, including stem cell factor (SCF) and interleukin-9 (IL-9) have been implicated in growth of normal MC, little is known about pro-oncogenic molecules and conditions triggering differentiation and growth of MC far enough to lead to the histopathological picture of overt mastocytosis. The anaplastic lymphoma kinase (ALK) has recently been implicated in growth of neoplastic cells in malignant lymphomas. Here, we describe that transplantation of *NPM-ALK*-transplanted mouse bone marrow progenitors into lethally irradiated IL-9 transgenic mice not only results in lymphoma-formation, but also in the development of a neoplastic disease exhibiting histopathological features of systemic mastocytosis, including multifocal dense MC-infiltrates, occasionally with devastating growth in visceral organs. Transplantation of *NPM-ALK*-transduced progenitors into normal mice or maintaintence of IL-9-transgenic mice without NPM-ALK each resulted in MC hyperplasia, but not in mastocytosis. Neoplastic MC in mice not only displayed IL-9, but also the IL-9 receptor, and the same was found to hold true for human neoplastic MC. Together, our data show that neoplastic MC express IL-9 rececptors, that IL-9 and NPM-ALK upregulate MC-production *in vivo*, and that both ‘hits’ act in concert to induce a mastocytosis-like disease in mice. These data may have pathogenetic and clinical implications and fit well with the observation that neoplastic MC in advanced SM strongly express NPM and multiple “lymphoid” antigens including CD25 and CD30.

## INTRODUCTION

Systemic mastocytosis (SM) is a neoplastic disease of hematopoietic progenitor cells [1-6]. The clinical and histopathological picture of the disease is characterized by abnormal growth and accumulation of mast cells (MC) in one or more visceral organs, with or without organ damage [1-8]. However, little is known about the pathogenesis and evolution of SM, and molecular mechanisms underlying the growth of neoplastic MC in affected organs. In the human system, the *KIT* proto-oncogene as well as the KIT-ligand stem cell factor (SCF), have been implicated in abnormal growth of MC in MC hyperplasia and SM [9-20]. Likewise, SCF reportedly induces differentiation of human MC *in vitro* [9, 10]. In addition, overexpression of endogenous SCF or injection of recombinant SCF is associated with a local increase in MC *in vivo*, which can closely resemble a primary MC disease (mastocytosis) [11-13]. In patients with SM, several gain-of-function mutations in *KIT* have been detected and supposedly contribute to autonomous growth of MC in SM [14-20]. However, not all patients with SM exhibit *KIT* mutations [7, 8]. Moreover, in other species, the contribution of KIT and the KIT-ligand SCF to the development of SM is less well established.

In mice, several different cytokines promote the development and differentiation of MC from their uncommitted bone marrow (BM) derived progenitors [21-28]. Among these cytokines are interleukin-3 (IL-3), IL-4, SCF, and IL-9 [21-28]. Especially SCF and IL-9 have been described to lead to MC hyperplasia when overexpressed or injected in mice [29, 30]. It has also been demonstrated that functional disruption of genes encoding IL-9, SCF, or SCF receptor (KIT), is associated with MC-deficiency [29-33]. In addition, several other factors and the microenvironment have been implicated in the regulation of growth and survival of (normal) murine MC [34-40]. However, so far, little is known about conditions and pro-oncogenic ‘hits’ required for full transformation of murine MC progenitors.

In previous studies, mutants of the *KIT* proto-oncogene have been described as regulators of growth of neoplastic MC and thus as a potential trigger in SM in mice [41-45]. However, apart from KIT, also other KIT-independent oncogenic molecules and triggers have been implicated in the development of mast cell tumors in mice [46-48]. Other studies have shown that ErbB can induce a mastocytosis-like disease in mice, and can substitute for KIT as MC growth-promoting kinase in *KIT*-deficient animals [49, 50]. However, little is known so far about the potential role and relative contribution of all these oncogenes and cytokines in growth of neoplastic MC in SM.

The anaplastic lymphoma kinase (ALK) is a src-receptor tyrosine kinase that is expressed in neoplastic B cells and T cells in several different lymphomas, but is not expressed in normal lymphohematopoietic cells [51-53]. In malignant lymphomas carrying the t(2;5)(p23;q35), *ALK* is usually fused to the *nucleophosmin (NPM)* gene [54-56]. We and others have shown that NPM-ALK leads to the formation of malignant lymphomas in mice [57-60]. In addition, it was found that transplantation of *NPM-ALK*-transduced BM cells into IL-9 transgenic mice results in the generation of various lymphoid and plasmacytoid tumors [61]. So far, however, the ALK tyrosine kinase and NPM have not been analyzed in the context of mastocytosis.

In this article, we show that the MC-targeting cytokine IL-9 and the lymphoma-related oncoprotein NPM-ALK act in concert to promote the growth of MC. In fact, whereas either ‘hit’ alone was found to lead to an increase in tissue MC in visceral organs in mice without fulfilling histopathological criteria of SM, transplantation of *NPM-ALK*-transfected progenitors into lethally irradiated IL-9 transgenic mice was found to be associated not only with lymphoma-formation, but also with the development of a neoplasm resembling systemic mastocytosis, sometimes with aggressive destructive growth indicative of advanced SM.

## MATERIALS AND METHODS

### Animals

FVB/N mice [62] were used as wild-type control and were purchased at 4–6 weeks of age from Jackson Laboratories (Bar Habor, ME). IL-9 transgenic mice were generated by microinjecting the IL-9 transgene-construct into the pronuclei of fertilized eggs of FVB mice as described [60, 63]. The homozygous transgenic mouse strain used in this study was designated Tg54. This strain maintained serum IL-9 levels of >1 μg/ml, while IL-9 was undetectable in serum of control FVB/N mice. Transgenic mice were generated at the Ludwig Institute for Cancer Research (Brussels, Belgium) and were bred and analyzed at the University of Schleswig-Holstein, Campus Lübeck (Lübeck, Germany). All animal work was performed according to local guidelines and international guidelines for animal care and protection. Animals were transplanted at 8–12 weeks of age.

### NPM–ALK retrovirus

A retroviral vector containing the *NPM-ALK* oncogene derived from a human NPM-ALK-positive anaplastic large cell lymphoma was cloned into the retroviral vector pLXSN to generate pL-NPM-ALK-SN [61]. In this vector, the 5′ viral LTR promoter/enhancer elements control the expression of the NPM-ALK gene. The SV40 early promoter (PSV40e) regulates the expression of the neomycin resistance gene (Neo^R^), which allows antibiotic selection in eukaryotic cells. Helper virus-free stocks of a vector control virus (mock virus) and the NPM-ALK-retrovirus were obtained from cloned ecotropic packaging cells GT+E86. This retroviral producer cell line, as well as NIH 3T3 cells infected with the virus, expressed the NPM–ALK protein as determined by RT-PCR and immunohistochemistry using an anti-ALK-1 antibody. Cells for retroviral infection were harvested from the marrow of the long bones of young TG54 and FVB/N mice and were cultured with infective supernatants [61].

### Transduction of BM cells and transplantation into irradiated recipients

BM cells were harvested from femurs of 8 to 12-week-old male IL-9 transgenic mice or FVB/N control mice, 6 days after these mice had been treated with 150 mg/kg 5-fluorouracil (Roche, Basel, Switzerland). Cells (5 × 10^5^ cells/ml) were cultured for 24 hours in DMEM (Invitrogen, Karlsruhe, Germany) supplemented with 10% fetal calf serum (FCS) (Invitrogen), recombinant mouse (rm) SCF (100 ng/ml), rmIL-6 (10 ng/ml), and rmIL-3 (10 ng/ml) (all from R&D Systems, Wiesbaden, Germany). Donor BM cells were transduced with L-NPM–ALK-SN virus supernatant with a multiplicity of infection (m.o.i.) of >5 at 37°C for 24 hours in DMEM with 10% FCS, rmSCF, rmIL-6, rmIL-3 and 5 mg/ml polybrene (Sigma Chemical Co., St Louis, MO, USA). After infection, BM cells were selected for 48 hours in medium complemented with G418^R^ (BD Biosciences Clontech, Heidelberg, Germany) and thereafter used for transplantation into lethally irradiated mice. One million cells were injected into one of the lateral tail veins of female recipient mice (8 to 10-week-old) after they had received a lethal dose of total body irradiation (5 Gy twice within a 3 hour-interval; 1.75 Gy/min; X-ray). Thereafter, mice received acidic water (pH 2.8) and sterile food, and were observed for the development of tumors. Three different experimental groups of mice were analyzed. In group ‘1’, a total of sixty two female IL-9 transgenic mice (TG54) were used and transplanted with L-NPM–ALK-SN-transduced BM cells from 10-week-old male IL-9 transgenic donors, resulting in fifty one IL-9/NPM-ALK double positive mice (11 mice died during the first 10 days after transplantation). Group ‘2’ contained a total of sixteen female IL-9 transgenic mice (TG54) and served as control group. These animals received LXSN-only infected BM cells, resulting in IL-9/control mice. In group ‘3’, a total of seventeen female wild-type mice (FVB/N) were transplanted with L-NPM-ALK-SN-transduced BM from 10-week-old male wild-type donors, resulting in NPM–ALK mice. A summary of animal groups examined is shown in Table 1. Ten female wild-type mice were transplanted with LXSN-only for control (negative for both IL-9 and NPM-ALK). In select experiments, 2 × 10^5^ lymphoma cells from tumor-bearing mice were obtained and injected intraperitoneally into syngeneic mice. Tumors developing in these secondary recipients were analyzed in the same way as primary tumors.

**Table 1.** Histologic patterns and neoplasms developing in wild type wt- and IL-9 transgenic mice transplanted with NPM-ALK-infected marrow or with mock infected control marrow

Mouse Species and Treatment	Lymphoblastic Lymphoma T-Cell Type	Plasmocytoma/Plasmoblastic Lymphoma	Mast cell Hyperplasia only^1^	Systemic Mastocytosis, small lesions	Systemic Mastocytosis, large lesions	Mast cell sarcoma or myeloid sarcoma
wt	0/2	0/2	0/2	0/2	0/2	0/2
IL-9+mock	4/16	0/16	12/16	0/16	0/16	0/16
wt+NPM/ALK	0/17	7/17	10/17	0/17	0/17	0/17
IL-9+NPM-ALK	17/51	33/51	10/51	10/51^2^	16/51^2^	4[1+3]/51^3^

The number of diseased animals is compared with the total number of animals included in each series of experiments. The majority of animals diagnosed to have mastocytosis also suffered from one or more malignant lymphomas.

1 In these mice, only mast cell hyperplasia but no mastocytosis-like disease developed.

2 Some of these mice developed both small lesions and larger lesions. One mouse developed a mast cell sacoma and presented with additonal SM lesions. In the “double hit” group, not all mice developed systemic mast cell disease – in fact, a mast cell disease was difficult to recognize when lymphomas developed very rapidly.

3 Of these 4 animals, 1 developed mast cell sarcoma/leukemia, and 3 a myelosarcoma (myelomonocytic sarcoma).

### Necropsy and histopathological examination of tissues sections

A complete necropsy was performed in each mouse. Moribund mice were euthanized and examined for evidence of tumor-formation. Thymus, lymph nodes, BM, spleen, stomach, gut, peyer's pathches and solid organs were analyzed by routine histology. Specimens were fixed by 4% paraformaldehyde (24 hours) before being embedded in paraffin. Tissue sections (3–5 μm thick) were placed on glass slides, dehydrated, dewaxed, and then stained with haematoxylin-eosin (H+E) or Giemsa solution. In addition, tissues sections were examined by toluidine blue- and Berberine sulphate staining. For toluidine blue staining, deparaffinized sections were stained in 1% toluidine blue (Merck; Darmstadt, Germany) dissolved in methanol (2-5 minutes), washed, and mounted after air-drying. For Berberine sulphate staining, deparaffinized sections were fixed in ethanol/acetic acid (3:1) for 15 minutes, washed in ethanol (15 minutes) and water (10 minutes), and then exposed to 0.02% berberine sulphate (Serva, Heidelberg, Germany) dissolved in water (pH 4 with 1% citric acid). Slides were then washed in water (pH 4.0 with 1% citric acid) for 5 minutes, air-dried, and mounted. Larger tumor lesions were dispersed and subjected to flow cytometry, or were snap frozen in liquid nitrogen for immunohistochemical analysis.

### Immunohistochemistry in mouse tissues

Affected mouse tissues were characterized immunophenotypically using the ABC-technique [59]. A panel of biotinylated first-step antibodies (all purchased from BD Bioscience, Heidelberg, Germany) was used: B-cell markers included CD45R, CD19, CD38 and CD23, and T-cell markers included CD5, CD25, CD4, CD8 and CD3e. For detection of these antigens, an alkaline phosphatase-conjugated streptavidin complex (Dako, Hamburg, Germany: K5005) was applied. For staining with mouse anti-human ALK-1 antibody M7185 (Dako) (heat retrieval at pH 9,5; work dilution: 1:25), anti-tryptase antibody M7196 (Dako) (heat retrieval at pH 9.5; dilution: 1:1,250), and anti-human KIT antibody (Labvision/Medac, Hamburg, Germany: K3954, applied according to the manufacturer's instruction), the Dako-ARK kit (animal research kit) was applied. The polyclonal antibodies anti-lambda, anti-kappa, anti-IgA, anti-IgG and anti-IgM, and secondary biotinylated goat anti-rabbit antibodies (all from Dako) were detected with goat anti-rabbit antiserum (Labvision/Medac; ultravision polymer, ready to use) according to the manufacturer's instructions. Microscopic evaluation and fotographic acquisitions were performed by using a MC200 chip microscope camera on a Zeiss Axioskop 2 plus microscope (Carl Zeiss GmbH, Jena, Germany) equipped with Planapochromat objectives (2.5x, 10x, 20x, 40x, 63x, 100x) and a numeric aperture of 1.4 using oil (Carl Zeiss GmbH). Images were obtained by using Adobe Photoshop CS2 software supply (Adobe, San Jose, CA) and processed with Power Point software, release 2003 (Microsoft, Redmont, WA).

### Evaluation of NPM-ALK mRNA expression by reverse transcription of RNA and quantitative PCR (qPCR)

Total RNA was extracted from 5 to 8 sections (25 μm thick) obtained from freshly frozen tissue blocks of mouse organs using Trizol reagent (Life Technologies GmbH, Karlsruhe, Germany). Total RNA was converted into cDNA using Superscript reverse transcriptase according to the manufacturer's recommendations (Life Technologies GmbH, Karlsruhe, Germany). Expression of mRNA specific for the human NPM-ALK fusion gene (accession no. U04946) was analyzed using cDNA populations derived from several organs and tumors of two transgenic mice (M780, M781) and one wild-type mouse (FVBN). The following primers were used: NAF (NPM-ALK-Forward Primer): AGCACTTAGTAGTGTACCGCCGGAAGCACC (breakpoint spanning) and NAR (NPM-ALK-Reverse Primer): TTCCATGAGGAAATCCAGTTCGTCC. The amplified product was 341 bp in size. NPM-ALK expression was compared to expression of the housekeeping gene mPBGD (accession no. NM_013551). The following primers were used to amplify a 428 bp mPBGD fragment: mPBGDF2: CAGACCGACACTGTGGTGGC (exon spanning) and mPBGDR2: CTTCCGAAGGCGGGTGTTGAG. Standard curves for NPM-ALK and mPBGD were generated from serial dilutions of cDNA (10^0^-10^4^). According to the equation E=10-1/slope the efficiency of amplification was calculated to be E=1.8 for NPM-ALK and E=1.95 for mPBGD. PCR reactions were prepared using the ‘LightCycler FastStart DNA MasterPLUS SYBR Green I kit’ and according to the instructions of the manufacturer. All samples were analysed in duplicates. PCR reactions were performed as follows: denaturing: 95°C; amplification (45 cycles): 95°C for 10 seconds, 55°C for 10 seconds, and 72°C for 25 seconds; melting: 65°C for 15 seconds; cooling: 40°C for 30 seconds. For quantification of NPM-ALK mRNA in tumor tissues, real time PCR was performed as described previously [64].

### Evaluation of IL-9 and IL-9 receptor (IL-9R) mRNA expression by reverse transcription of RNA and qPCR

For detection and quantification of IL-9 mRNA and IL-9 receptor mRNA in mouse tissues and human neoplastic mast cells (HMC-1), standard PCR protocols were applied. Prior to RNA isolation and qPCR, HMC-1 cells were incubated with 1 μg polysaccaride (LPS, Sigma) in 10 ml medium for 4 hours. The qPCR was carried out using the LightCycler FastStart DNA Master Plus SYBR Green I Kit, according to the manufacturer's instructions (Roche). For each single reaction, 3 μl of cDNA was used. The reaction was initiated with a 10 minute-denaturation step at 95°C, and terminated with a 30 second cool-step at 40°C. The cycling protocol consisted of a denaturation step at 95°C for 10 seconds, annealing at 56°C (10 seconds), and extension step at 72°C for 25 seconds, and was repeated 45 times. Ramp time for all steps was 20°C/second. Relative quantification was performed by the 2-delta-delta-C(T) method employing β-actin as a housekeeping gene as described [65]. For PCR the Pure Taq Ready-To-Go PCR beads system of GE Healthcare (Cat. No. 27-9559-01) was used. Beads were supplemented with 10 pmol of each primer, DNA and HPLC grade H_2_O to a final volume of 25 μl. Each PCR cycle consisted of 30 seconds denaturation at 94°C, 30 seconds annealing at 56 °C, and 90 seconds elongation at 72°C, and was repeated 30 times. Initial denaturation was performed for two minutes at 94°C, and final elongation for seven minutes at 72°C. Electrophoresis was performed on a 2% agarose gel. Primers used in qRT- and PCR experiments are shown in Table 2.

**Table 2.** PCR Primers

Primers - Mouse	Forward primer (For)	Reverse primer (Rev)
mIL9 (NM_008373)	For: 5′-tgattgtaccacaccgtgct-3	Rev: 5′-gcttttctgcctttgcatct-3′
mIL9R (NM_008374)	For: 5′-atgggacaggaacaggtcag-3′	Rev: 5′-aggtcactccaacgatacgg-3′
beta-mActin (NM_001101)	For: 5′-tgacgaggcccagagcaagagag-3′	Rev: 5′ctaggagccagagcagtaatctg-3′
**Primers - Human**		
hIL9 (NM_000590)	For: 5′-tctgacaactgcaccagacc-3′	Rev: 5′-ttgcctctcatccctctcat-3′
hIL9R1 (NM_002186)	For: 5′-agctatgagctggccttcaa-5′	Rev: 5′-ccacatcatcctccagtgtg
hIL9R2 (NM_176786)	For: 5′-agctatgagctggccttcaa-3′	Rev: 5′-ccacatcatcctccagtgtg-3′

Primer pairs were designed according to published sequences for mouse and human genes.

### Culture of HMC-1 cells and stimulation experiments

The human mast cell line HMC-1, established from neoplastic MC of a patient with MC leukemia [66] was kindly provided by Dr. J.H. Butterfield (Mayo Clinic, Rochester, MN). HMC-1 cells were grown in Iscove's modified Dulbecco's medium (IMDM) (Gibco Life Technologies, Gaithersburg, MD) with 10% FCS (PAA laboratories, Pasching, Austria), L-glutamine, and antibiotics at 37°C and 5% CO2. Two subclones of HMC-1 were used, namely HMC-1.1 harbouring the KIT mutation V560G but not *KIT* D816V, and a second subclone, HMC-1.2, harbouring *KIT* V560G and D816V. HMC-1 cells were re-thawed from an original stock every 4-8 weeks and were passaged weekly. As control of ‘phenotypic stability’, HMC-1 cells were periodically checked for expression of surface KIT and the down-modulating effect of IL-4 (100 U/ml, 48 hours) on KIT-expression. In stimulation experiments, HMC-1 cells were cultured in the absence or presence of LPS (0.1 μg/ml) for 4-24 hours (37°C), or in the absence or presence of recombinant human IL-9 (5-100 ng/ml) (R&D systems, Wiesbaden, Germany) for 72 hours (37°C). After exposure to IL-9, uptake of 3H-thymidine was determined. All experiments were performed in triplicates.

### Flow cytometry

Direct immunofluorescence staining of dispersed mouse tissue cells was performed using phycoerythrin (PE)-conjugated rat monoclonal antibodies (mAb) against B-cell antigens (CD45R, CD19, CD38 and CD23), T-cell antigens (CD5, CD25, CD4, CD8 and CD3e), and myeloid antigens (CD11c, CD34) (all from BD Bioscience). HMC-1 cells were analyzed by flow cytometry using PE-conjugated mAb 104D2 (IgG1) directed against KIT (Becton Dickinson), mAb 33423 (IgG1) directed against the human IL-9 receptor (R&D systems), and an isotype-matched IgG1 control antibody. The anti-IL-9 receptor antibody was detected by a second step FITC-labeled goat anti-mouse IgG antibody (R&D systems). Mouse cells and HMC-1 cells (each 10,000 per test) were analyzed on a FACScan (BD Bioscience). All staining reactions were controlled by isotype-matched antibodies.

### Examination of primary neoplastic MC for expression of NPM, ALK, IL-9 and IL-9 receptor by immunohistochemistry

In 19 patients (age 26-63 years) with SM, immunohistochemistry was performed on sections obtained from formalin-fixed and paraffin-embedded BM samples. Informed consent was obtained in each case before BM biopsy. Of the 19 patients, 7 suffered from indolent SM (ISM), 2 from smouldering SM (SSM), 4 from SM-AHNMD, 3 from aggressive SM (ASM), and 3 from mast cell leukemia (MCL). Expression of tryptase, CD25, CD30, NPM, and ALK were analyzed in BM sections by indirect immunohistochemistry using a published protocol [67] and the following antibodies: anti-tryptase mAb G3 (Santa Cruz Biotechnology, Santa Cruz, CA, 1:500), anti-CD25 mAb 4C9 (Novocastra, Newcastle upon Tyne, UK), anti-CD30 mAb Ber-H2 (Dako, Glostrup, Denmark; 1:20), mAb 376 against nucleophosmin (Dako; 1:800), and anti-ALK mAb ALK1 (Dako; 1:25). 3,3-diaminobenzidine (DAB) was used as chromogen. Slides were counterstained in Mayer's Hemalaun. For detection of IL-9 and IL-9R, a three step biotin-free polymer detection method was used after heat-induced epitope retrieval, according to the protocol provided by the manufacturer (UltraVision LP Detection System HRP Polymer & DAB Plus Chromogen, Labvison, Thermo-Fisher, provided by Medac, Hamburg Germany). The dilutions of the antibodies used were 1:200 for rabbit anti-IL-9 (R&D Systems), and 1:400 for mouse anti-IL-9R mAb (R&D Systems). Horseradish peroxidase and 3,3′ diaminobenzidine/H_2_O_2_ were applied yielding a brown color reaction. Hematoxylin was used as counter-stain. The photomicrographs were obtained using a Zeiss Axiophot microscope (Carl Zeiss, Jena, Germany).

## RESULTS

### Retroviral transfer of *NPM-ALK* into primary mouse BM cells and detection of NPM-ALK mRNA in infected cells after transplantation

**Figure 1.**
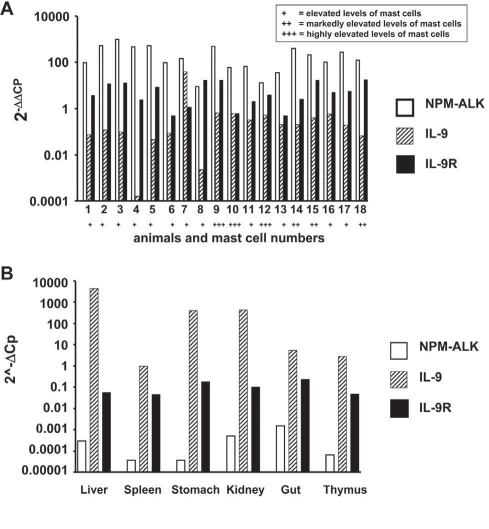
Expression of NPM-ALK mRNA, IL-9 mRNA, and IL-9 receptor mRNA in mouse tissues (A) After retroviral overexpression of NPM-ALK in IL-9 transgenic mice, an increase in mast cells in various organs was found, which is indicated by the following score (bottom line on x axis): +, elevated levels of mast cells; ++, markedly elevated levels of mast cells; +++, highly elevated levels of mast cells. In the same organs, expression of NPM-ALK mRNA (open bars) as well as IL-9 mRNA (hatched bars) was demonstrable by qPCR. A series of 18 animals is shown. The following organs were analyzed in these animals: thymus (1), liver (2), spleen (3), lung (4), gut (5), kidney (6), gut (7), spleen (8), spleen (9), liver (10), kidney (11), gut (12), stomach (13), spleen (14), stomach (15), stomach (16), stomach (17), and thymus (18). qPCR was performed as described in the text. Interestingly, we were also able to demonstrate expression of significant amounts of IL-9 receptor (IL-9R) mRNA (black bars), probably expressed in mast cells, in all tissues and organs examined. B: Expression of NPM-ALK mRNA levels, IL-9 mRNA levels, and IL-9 receptor (IL-9R) mRNA levels in various organs in an IL-9 transgenic mouse transplanted with Neo+(wt)-transfected cells. As visible, IL-9 transcripts were detected in all organs, whereas NPM-ALK mRNA was virtually undetectable. Interestingly, in IL-9 transgenic mice (without NPM-ALK expression), we also were unable to detect substantial levels of IL-9 receptor mRNA by qPCR. In control mice, neither IL-9 nor IL-9 receptor mRNA or NPM-ALK mRNA was detected (not shown).

In four independent experiments, 51 IL-9 transgenic mice with NPM-ALK+ BM cells and 16 IL-9-transgenic mice with Neo+ (wt) BM cells were analysed. NPM-ALK+ BM cells were transplanted into 17 FVB/N wild-type mice (not transgenic for IL-9), which served as control. As assessed by real-time PCR [64, 65], NPM-ALK mRNA was found to be expressed in all tumors in IL-9 transgenic mice transplanted with pL-NPM-ALK-SN-transfected cells (Figure 1A), but not in IL-9 transgenic mice transplanted with Neo+(wt)-transfected cells (Figure 1B). In mice transplanted with pL-NPM-ALK-SN-transfected cells, the levels of NPM-ALK mRNA varied slightly from tissue to tissue and from tumor to tumor. Nevertheless, significant levels of NPM-ALK mRNA were detectable in all tissues (organs) affected with mastocytosis regardless of the presence or absence of a lymphoma, and without differences in NPM/ALK mRNA levels among animals examined (Figure 1A).

### Both NPM-ALK and IL-9 induce mast cell hyperplasia in mice

As assessed by immunohistochemistry of BM sections and tissue sections in other organs, a slight to moderate increase in mast cells was found in IL-9 transgenic mice as well as in FVB/N (IL-9-negative) mice transplanted with pL-NPM-ALK-SN-transfected cells, but not in FVB/N control mice transplanted with pLXSN-transduced (neo+) BM cells (Table 1). These data suggest that both IL-9 as well as NPM-ALK promote growth and/or differentiation of MC (progenitors) *in vivo.* Regarding IL-9, these data are consistent with the notion that IL-9 is an MC growth factor in mice [27]. However, under the experimental conditions applied, neither IL-9 nor NPM-ALK alone (as single “hit”) were found to lead to the histopathological picture of mastocytosis (focal MC accumulations resembling SM) in any animal and organ examined (BM, spleen, liver, lung, thymus, gastrointestinal tract) (Table 1).

### NPM-ALK and IL-9 cooperate in producing a mastocytosis-like disease in mice

**Figure 2.**
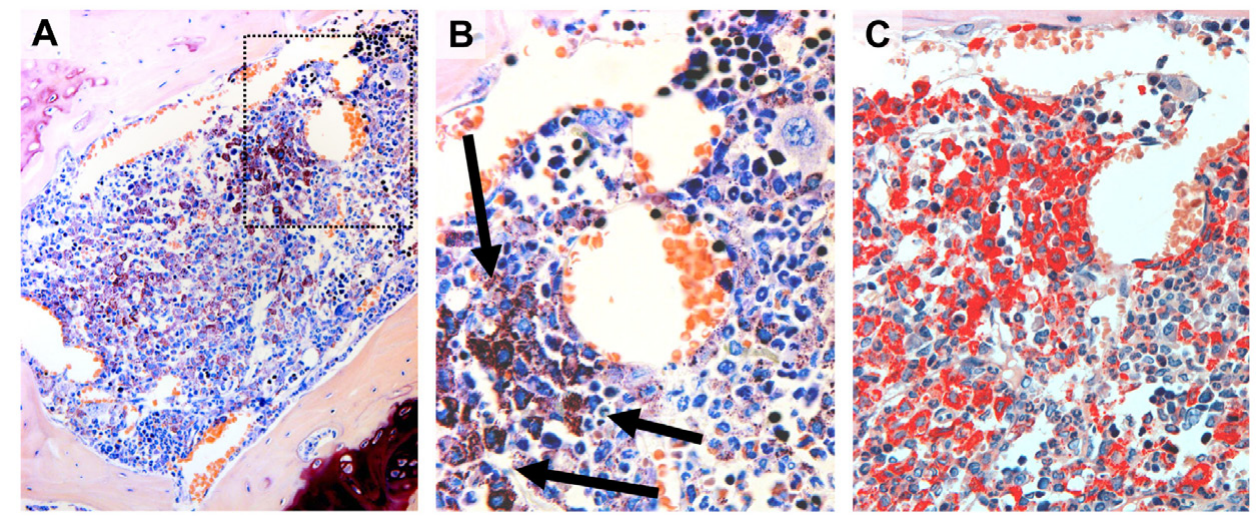
Mastocytosis in the bone marrow in an NPM-ALK-positive IL-9 transgenic mouse The generation of IL-9 transgenic mice, transplantation of NPM-ALK positive progenitor cells, tissue processing, and staining techniques are described in the text. (A, B): Giemsa-stained dense mast cell infiltrate in the bone marrow of an NPM-ALK-positive IL-9 transgenic mouse is shown (magnification, A, 630x; B, 1000x). The higher magnification (B) shows that most mast cells (arrows) were round cells with moderately or well granulated cytoplasm and a round nucleus indicating an indolent variant of mastocytosis. Correspondingly, these mast cells were found to stain positive for Naphthol AS-D chloroacetate esterase (CAE) (x630) (C).

**Figure 3.**
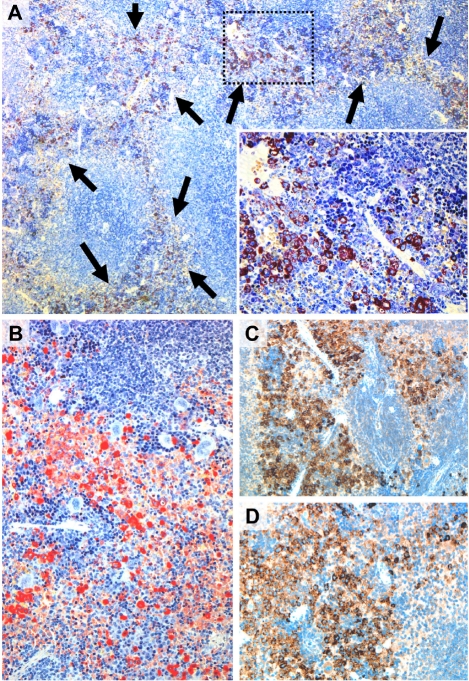
Mastocytosis infiltrates in the spleen in NPM-ALK-positive IL-9 transgenic mice The generation of IL-9 transgenic mice, transplantation of NPM-ALK positive progenitor cells, tissue processing, and staining techniques are described in the text. (A) shows a Giemsa-stained section of the spleen in an IL-9 transgenic, NPM-ALK-positive mouse (magnification, 100x, insert 400x). The splenic tissue is markedly infiltrated by mast cells (arrows) with subtotal replacement of the pre-existing red pulp. The white pulp is still preserved. Most mast cells were found to be round cells and to contain numerous metachromatic granules in their cytoplasm. These mast cells were also found to stain positive (red color) for Naphthol AS-D chloroacetate esterase (CAE) (100x) (B) and to co-express tryptase (C) (x200) and CD25 (x200) (D) as assessed by immunohistochemistry (ABC method). For details of staining protocols see text in Materials and Methods.

**Figure 4.**
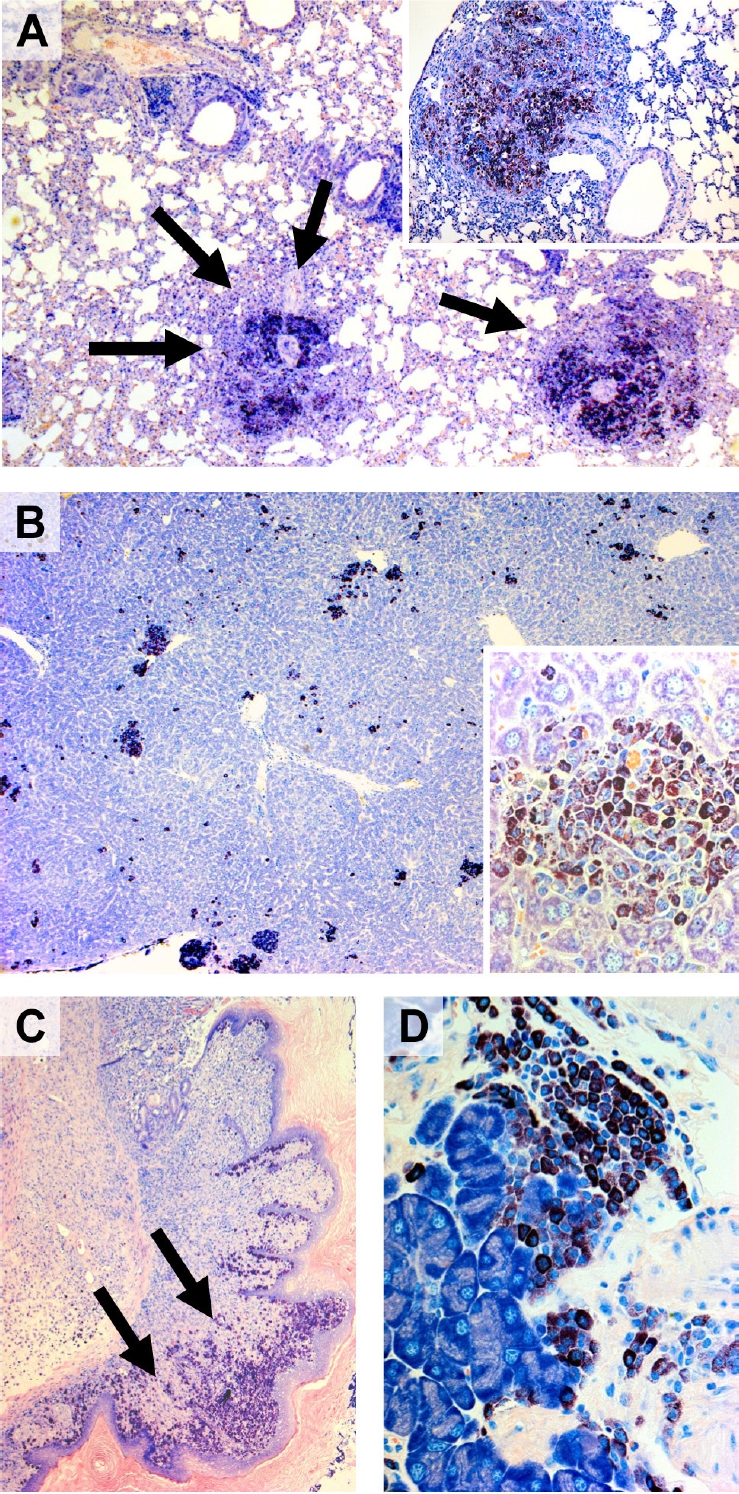
Mast cell infiltrates in various extramedullary organs in NPM-ALK-positive IL-9 transgenic mice Tissue sections of various organs obtained from NPM-ALK-positive IL-9 transgenic mice were stained by Giemsa. As visible, mast cell infiltrates (arrows) were detected in the lungs (A) (overview: magnification x20; insert x100), the liver (B) (overview x20; insert x400), the gastrointestinal tract (C, showing the esophago-gastric junction, x20), and the pancreas (D) (pancreatic gland, x400). In most instances, mast cells were found to be rather mature and well granulated cells consistent with the diagnosis of an indolent variant of a systemic mast cell disease. Some of these cells, however, were found to be more immature with a prominent nucleus, nucleoli, and a hypogranulated cytoplasm.

All IL-9-transgenic mice that had received NPM–ALK-transduced hematopoietic precursor cells developed hematopoietic malignancies, including various types of lymphomas (Tables 1 and 3). Tumor development was detected within 30 weeks. Macroscopically, most mice developed prominent abdominal lymph node masses, including peripancreatic lymph nodes, retroperitoneal lymph nodes, and splenomegaly. Most of these tumor lesions were found to contain lymphoma cell infiltrates, confirming previous data [61]. In 4 cases, immature myeloid neoplasms (resembling myelosarcoma or MC sarcoma/leukemia) were detected (Tables 1 and 3). Most significantly, however, we found that apart from lymphomas, substantial accumulations of MC in various organs developed in a significant number of (27/51) animals (Tables 1 and 3). In a subgroup of these animals (n=15), larger lesions of MC with typical clusters or even sheets of MC were found. IL-9/NPM-ALK+ mice with no apparent accumulations of MC (n=15) died early, some during the acute period after transplantation (marrow aplasia), others from aggressive lymphomas, suggesting that MC neoplasms developed later than lymphomas in mice. Infiltrates of neoplastic MC were found in various organs and tissues including the BM (Figure 2), the spleen (Figure 3), liver (Figure 4), lymph nodes, thymus, gastrointestinal tract (stomach and gut) (Figure 4), lung (Figure 4), and kidney. In most animals, MC accumulations were found to resemble an MC neoplasm (mastocytosis) with dense focal MC aggregates (Figure 2-4). MC infiltrates were found either as an isolated lesion (without lymphoma) or together with a co-existing lymphoma-lesion at the same tissue site.

**Table 3.** Organs involvement with mast cell disease/accumulation in IL-9-transgenic NPM-ALK+ mice

Experiment	MC-accumulation x/n mice	Organ involvement by substantial mast cell accumulations	Main histologic diagnosis
#1	1/10	spleen, stomach	SM + pc
#2	6/10	BM, LN, spleen, kidney, lung, stomach, gut	SM + pbl
		LN, spleen, kidney, lung, stomach, gut	SM + pc
		kidney, heart, lung	SM + pc
		liver, lung, stomach, thymus	SM + pc + mastsrc
		BM, LN, spleen, liver, lung, stomach, gut	SM + pc + mysarc
		LN, spleen, lung, stomach, gut	SM + pc
#3	6/14	spleen	SM + pc
		spleen, liver, lung, stomach	SM + pc
		spleen, stomach, gut	SM + pc
		LN, spleen, stomach	SM + pc
		BM, spleen, lung, stomach, gut	SM + pc + T-lb
		BM, LN, spleen, lung, stomach, gut	SM + pc
#4	14/17	spleen, liver, lung, stomach, thymus, gut	SM + pc + pbl + T-lb
		BM, spleen, liver, lung	SM + pc
		LN, spleen, liver, kidney, lung, stomach, gut	SM + pc
		spleen, liver, gut	SM + pc + T-lb
		BM, spleen, liver, lung	SM + pbl
		LN, lung, stomach	SM + T-lb
		liver, heart, lung, gut	SM + pc
		BM, spleen, stomach	SM + pc + T-lb
		BM, lung, stomach	SM + T-lb + mysarc
		LN, spleen, thymus, gut	SM + T-lb
		LN, liver, lung, stomach	SM + T-lb
		spleen, liver, lung	SM + T-lb
		BM, LN, spleen, liver, kidney, thymus	SM + T-lb
		spleen, liver, stomach	SM + pc + T-lb
Total	27/51		

Results are derived from a complete necropsy and histopathological as well as immunohistochemical analysis of tissue sections in IL-9 transgenic mice transfected with NPM-ALK retrovirally-transformed bone marrow cells. Only mice in which malignant mast cell lesions had developed are shown. BM, bone marrow; LN, lymph nodes; SM, systemic mastocytosis; T-lb, T lymphoblastic lymphoma; pc, plasmocytoma; pbl, plasmoblastic lymphoma; mysarc, myelosarcoma, mastsrc, mast cell sarcoma.

### Histology of mast cell infiltrates in IL-9 transgenic NPM-ALK+ mice

MC accumulations could easily be detected by Giemsa-staining in tissue sections in IL-9 transgenic NPM-ALK+ animals (Figure 2-4). In a few animals, some of the lesions showed a more diffuse infiltration pattern resembling MC hyperplasia rather than true mastocytosis (Table 1). In a majority of the animals with advanced MC infiltration, however, most lesions showed dense compact clusters of rather mature and moderately or even well granulated MC (Figures 2-4). In most of these animals, the lesions did not show histological signs of aggressive disease or a devastating growth pattern (Figures 2-4). In a few animals, however, MC aggregates extended to more confluent sheets of MC with associated organ fibrosis or even signs of destructive growth, consistent with the diagnosis of an aggressive MC disease (n=15/51) or even mast cell sarcoma (n=1/51). A summary of MC disorders detected in mice is shown in Tables 1 and 3. In general, MC lesions were found in most visceral organs in IL-9 transgenic NPM-ALK+ mice. In several animals, MC aggregates were also found in the lungs (Figure 4A). Occasionally, the kidneys or the thymus were found to be infiltrated by neoplastic MC. In one case, the thymus was destroyed by a large MC infiltrate exhibiting a sarcoma-like growth pattern. A summary of involved organs in affected IL-9 transgenic NPM-ALK+ mice is shown in Table 3.

### Morphology and histochemical characteristics of mast cells in IL-9 transgenic NPM-ALK+ mice

The morphology of MC varied from animal to animal and also from lesion to lesion, depending on the type of disease. In fact, MC often appeared to be rather mature and well granulated cells in MC hyperplasia and in animals exhibiting mastocytosis with small clusters and aggregates of MC (Figures 3 and 4). However, in animals developing aggressive MC disease, the morphology of neoplastic MC appeared to be immature, often with a hypogranulated cytoplasm and sometimes with bi- or polylobed nuclei. In all IL-9 transgenic NPM-ALK+ mice examined, neoplastic MC were found to stain positive for Giemsa and toluidine blue as well as Berberine sulfate (not shown), independent of the type of disease and organ/tissue site examined. We also found that neoplastic MC clearly stained positive for Naphthol AS-D chloroacetate esterase (CAE) (Figure 2C). Together, as assessed by morphology and cytochemistry, MC in most IL-9 transgenic NPM-ALK+ mice were found to resemble mature tissue MC. In mice with progressive MC lesions, however, MC were found to be immature hypogranulated cells, which is consistent with observations in human SM [7, 8].

### Immunohistochemical properties of mast cells in IL-9/NPM-ALK+ mice

In a first step, we confirmed that neoplastic MC in IL-9 transgenic NPM-ALK-induced mastocytosis indeed expressed the NPM-ALK protein. For this purpose, ALK-1 expression was examined by immunohistochemistry using the ARK-kit, a technique in which the mouse antibody (ALK1) becomes saturated with biotinylated anti-mouse-Fab fragment to reduce anti-mouse Ig-reactions and thus false-positive reactions through Fc-binding [61]. Using this technique, we were able to show that neoplastic MC in all affected tissues in all NPM-ALK+ mice invariably expressed the ALK protein (Table 4). Lymphoma cells also expressed the ALK protein in all NPM-ALK mice analyzed (Table 4), whereas the unaffected tissues in these animals were found to be ALK-negative. MC in MC hyperplasia in IL-9 transgenic mice (NPM-ALK-negative) did not stain positive with the ALK-antibody (not shown). In a second step, we examined the antigen profile of neoplastic MC in IL-9 transgenic NPM-ALK+ mice. As expected, these MC stained positive for CD45, CD117 (KIT), MC tryptase (Figure 3C), and MC chymase (Table 4). An interesting aspect was that these MC also expressed CD25 (Figure 3D, Table 4), a marker that is typically expressed in neoplastic MC in human SM. Neoplastic MC did not express (other) T cell- or B cell antigens (CD3, CD4, CD5, CD8, CD19) (Table 4). We were also unable to detect CD23, CD38, or the progenitor antigen CD34 in neoplastic MC of these mice (Table 4). Vice versa, neoplastic cells in T cell- or B cell-lymphomas did not express MC-related antigens (KIT, tryptase, chymase) in any of the animals examined. A summary of staining results obtained in neoplastic cells in IL-9/NPM-ALK+ mice is shown in Table 4.

**Table 4.** Expression of lymphohematopoietic differentiation antigens in neoplastic cells in IL-9/NPM-ALK+ mice

Antigen/CD	Reactivity of cells with antibodies in			
	Lymphomas		Mastocytosis mast cell tumor	Myelosarcoma
	T-type	pc/pb		
TCR/CD3	+/−	−	−	−
T4/CD4	+/−	−	−	−
CD5	+	−	−	−
T8/CD8	+/−	−	−	−
CD19	−	+/−	−	−
FceRII/CD23	−	+/−	−	−
IL-2Rß/CD25	+/−	−	+	−
CD34	−	−	−	−
CD38	−	+	−	−
LCA/CD45	+	+/−	+	+/−
KIT/CD117	−	−	+	−
Tryptase	−	−	+	−
Chymase	−	−	+	−
ALK	+	+	+	+/−

Expression of leukocyte differentiation antigens in neoplastic cells was determined by immunohistochemistry and/or flow cytometry. With both techniques, identical results were obtained. LCA, leukocyte common antigen; ALK, anaplastic lymphoma kinase. +, expressed in all cells; +/−, expressed in a subpopulation or weak expression; -, not expressed.

### Separation of lymphomas from mast cell tumors by transfer experiments

To study the (common) origin of lymphoma stem cells and mast cells in individual animals, transfer experiments were performed using isolated cells derived from lymphoma lesions of IL-9/NPM-ALK+ mice. After intraperitoneal injection of tumor cells, recipient mice (control background = IL-9-negative or IL-9 transgenic mice) developed tumours within 4–10 weeks. The resulting neoplasms were invariably classified as lymphomas by histology and immunophenotyping, with tumor-subtypes resembling the same histology as that of the injected tumors. However, no MC tumors developed in any of the secondary recipient mice examined. These data suggest that NPM-ALK transforms two distinct types of neoplastic stem cells in mice, i.e. a lymphoma stem cell and a MC stem cell, and that these two populations of cells grow independent from each other. Unfortunately, we were unable to perform experiments to generate a mastocytosis-like disease in secondary recipient mice, because it was not possible to predict whether the explanted organ would contain sufficient numbers of neoplastic MC (mastocytosis was rarely associated with tumor formation). In addition, lymphomas (either T-lymphoblastic or B-plasmocytic/blastic) were highly aggressive and thus had a growth advantage over slowly growing MC lesions.

### Detection of IL-9 mRNA and IL-9 receptors in IL-9 transgenic NPM-ALK+ mice

Based on our mouse model, IL-9 is considered to play a potential role in the development of MC hyperplasia/mastocytosis. Therefore, we were interested to learn whether mouse tissue samples would express IL-9 or IL-9 receptor mRNA. As expected, neoplastic cells in the tumors obtained from IL-9 transgenic mice expressed IL-9 mRNA (Figure 1). In addition, we were able to detect IL-9 receptor mRNA in these cells, although the transcript levels were rather low (Figure 1).

### Detection of IL-9 mRNA and IL-9 receptors in the human mast cell leukemias cell line HMC-1

**Figure 5.**
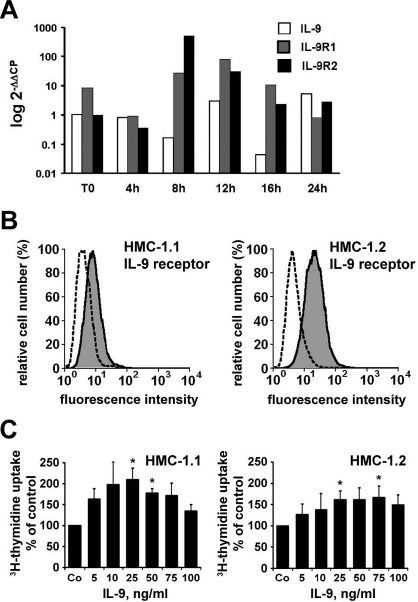
Expression of IL-9 and IL-9 receptors in HMC-1 cells A: Expression of IL-9 mRNA, IL-9R1 mRNA, and IL-9R2 mRNA in HMC-1.2 cells after stimulation with lipopolysaccharide (LPS). HMC-1 cells were incubated with LPS (0.1 μg/ml) for various time periods (4-24 hours = h) as indicated. Thereafter, cells were analyzed for expression of IL-9 mRNA, IL-9R1 mRNA, and IL-9R2 mRNA by qPCR using primers depicted in Table 2. In the absence of LPS, baseline transcript levels did not change at any time point compared to time point “0” (not shown). B: Expression of surface IL-9 receptor on HMC-1.1 cells (left panel) and HMC-1.2 cells (right panel) as determined by flow cytometry. Cells were labelled with an anti-IL-9 receptor antibody (grey histogram) or an isotype-matched control antibody (stippeled histogram) by indirect immunofluoresence staining. C: Effects of IL-9 on growth of HMC-1.1 cells (left panel) and HMC-1.2 cells (right panel). HMC-1 cells were incubated in control medium (Co) or various concentrations of IL-9 (as indicated) for 72 hours at 37°C. Thereafter, 3H-thymidine uptake was measured. Results represent thymidine uptake as percent of control and show the mean±S.D. of three independent experiments. Asterisk (*) indicates p<0.05 compared to control (Co).

HMC-1 cells were found to exhibit low levels of IL-9 mRNA and low but detectable levels of IL-9 receptor mRNA as unstimulated cells. When exposed to LPS, HMC-1 cells were found to display higher levels of IL-9 receptor mRNA (Figure 5A). Surprisingly, IL-9 mRNA levels even decreased after LPS treatment of HMC-1 cells (Figure 5A). We next explored whether HMC-1 cells also express the IL-9 receptor on their cell surface. As assessed by flow cytometry, we were indeed able to show that both HMC-1 subclones examined, i.e. HMC-1.1 (lacking KIT D816V) and HMC-1.2 cells (expressing KIT D816V) express the IL-9 receptor on their surface (Figure 5B). Finally, we asked whether IL-9 would promote the growth of HMC-1 cells *in vitro.* As visible in Figure 5C, IL-9 was found to promote the growth of HMC-1.1 cells and HMC-1.2 cells in a dose-dependenmt manner. These data suggest that neoplastic human MC, i.e. HMC-1 cells, express functional receptors for IL-9.

### Examination of primary neoplastic human MC for expression of IL-9 and IL-9 receptors

**Figure 6.**
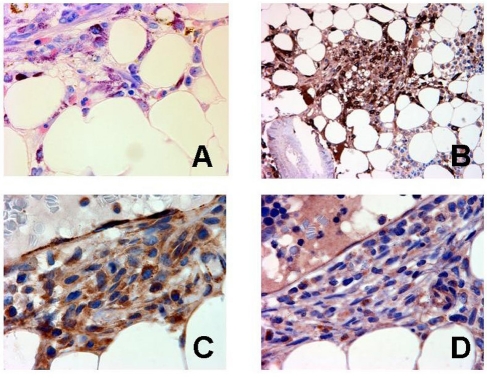
Expression of IL-9 and IL-9 receptor in primary human neoplastic mast cells (MC) Bone marrow sections obtained from a patient with systemic mastocytosis were stained with Giemsa (A) (magnification, x630), an antibody against tryptase (B) (x400), an antibody against the IL-9 receptor (C) (x1,000), and an antibody against IL-9 (D) (x1,000). The staining protocols are provided in the text. As visible, neoplastic MC were found to be immunoreactive for both IL-9 and the IL-9 receptor.

To demonstrate expression of IL-9 and IL-9 receptors in primary human neoplastic MC in SM, a cohort of patients with human SM were examined using routine bone marrow sections and immunohistochemistry. Immunohistochemi cal analysis on serial sections revealed strong expression of IL-9 receptors in neoplastic (tryptase+) MC in all SM cases examined. A typical example is shown in Figure 6. MC in normal healthy individuals also expressed IL-9 receptors (data not shown). In addition, we were able to show that neoplastic MC express IL-9 (Figure 6D). However, whereas the IL-9 receptor was detected in MC in all cases examined, IL-9 was expressed (weakly) in only 3/7 patients analyzed. IL-9 was also found to be expressed by Hodgkin- and Sternberg-Reed cells in cases of Hodgkin's lymphomas, which were employed as a positive control for IL-9 staining (not shown). In a final step, we asked whether neoplastic MC in SM express ALK or other lymphoma-related antigens in serial BM sections. In these experiments, we were able to show that tryptase+ MC co-express CD25 as well as nuclear NPM but did not express ALK, independent of the variant of SM (Table 5). An interesting observation was that MC in aggressive SM expressed substantial amounts of NPM, sometimes even in their cytoplasm, whereas in ISM, NPM was only expressed weakly in MC, and was only detectable in the nuclei of neoplastic MC (Table 5). Confirming our previous observations [68, 69], CD30 was found to be expressed in MC in ASM and MCL, whereas in ISM, most MC usually stained negative for CD30 (Table 5).

**Table 5.** Expression of lymphohematopoietic antigens in neoplastic MC in human SM

Marker/Antigen	Expression of antigen detected in BM mast cells by IHC* in			
	ISM	SSM	ASM	MCL
Tryptase	+	+	+	+
CD117/KIT	+	+	+	+
CD25	+	+	+	+
CD30	−	+/−	+	+
NPM(n)	+/−	+	+	+
NPM(c)	−	−	+/−	+/−
ALK	−	−	−	−

Abbreviations: MC, mast cells; SM, systemic mastocytosis; BM, bone marrow; IHC, immunohistochemistry; ISM, indolent SM; SSM, smouldering SM; ASM, aggressive SM; MCL, mast cell leukemia; NPM(n), nuclear nucleophosmin; NPM(c) cytoplasmic nucleophosmin.

* Expression of antigens in BM MC was determined on serial BM sections by IHC using tryptase as MC-specific marker.

## DISCUSSION

Several lines of evidence support the ‘multi-hit’ concept of cancer development [70-72]. This concept is well established for various types of lymphohematopoietic neoplasms in man and mice [71, 72]. However, although many different factors and genes have been implicated in the regulation of growth of normal MC, little is known about oncogenic ‘hits’ that can act in concert to transform MC progenitors and thus lead to the clinical picture of overt mastocytosis. In the current study, we show that IL-9 and NPM-ALK each promote the production of MC *in vivo*, and that both act in concert to induce a mastocytosis-like disease in mice. In fact, IL-9 transgenic mice transplanted with NPM-ALK-transfected BM cells developed not only ALK+ lymphomas, but also various types of benign and malignant MC lesions. Based on their morphology, histology, phenotypic properties, and on transfer experiments, MC neoplasms and ALK+ lymphomas could clearly be distinguished in these mice.

A number of different cytokines including SCF, IL-3, IL-4, IL-9, and IL-13 have been described to act as MC differentiation- and growth factors in mice [21-28]. Correspondingly, we were able to detect MC hyperplasia in most of our IL-9 transgenic mice, thereby confirming earlier observations [30]. However, these IL-9-triggered accumulations of MC did by far not reach criteria for a primary MC disease (MC neoplasm=mastocytosis) in any of the animals examined.

The observation that NPM-ALK promotes the production of MC, with resulting MC hyperplasia in various mouse tissues, was an unexpected result. In fact, NPM-ALK has been described as a lymphoma-specific defect [51-53], but has not been implicated in the development of MC hyperplasia or an MC neoplasm so far. In addition, normal tissues and cells including myeloid progenitors in the BM, usually do not express ALK [54-56]. The unexpected effect of NPM-ALK on MC progenitors may have several explanations. First, this oncogene may target a very immature stem cell capable of giving rise to lymphoid and myelomastocytic progenitors. This hypothesis would be supported by the concordant development of lymphomas and MC neoplasms in mice, and by the occurrence of very immature hematopoietic (sometimes myelosarcoma-like) neoplasms in some of these animals. It is noteworthy in this regard that NPM-ALK is a tyrosine kinase [53-55] and that several other tyrosine kinases can regulate growth of MC in mice [49, 50]. It is also noteworthy that NPM-ALK and KIT (as well as other tyrosine kinase receptors) may utilize similar downstream signaling pathways regulating growth and survival of neoplastic cells [53-55]. Another explanation for the MC-promoting effect of NPM-ALK would be that malignant cells in the NPM-ALK-induced lymphomas produced cytokines that induced MC growth, such as IL-3 or IL-4. In this regard it is noteworthy that the NPM-ALK-induced lymphomas were often found to be of T cell origin, and that T cells usually produce and release huge amounts of such MC-targeting cytokines [21-23]. A third explanation for the MC-promoting effect of NPM-ALK may be a specific effect of the NPM-component of the oncoprotein. Notably, it has been described that NPM is mutated and may play a pathogenetic role in other myeloid neoplasms, such as acute myeloid leukemia [73]. Furthermore, we were able to show in this study, that neoplastic MC in human SM express NPM, and that NPM-expression may correlate with the variant of SM. However, human neoplastic MC in SM did not express ALK, and similar to IL-9, NPM-ALK alone led to MC hyperplasia only, but did not lead to the clinical picture of overt mastocytosis.

The observation that transplantation of NPM-ALK-transfected progenitors into IL-9 transgenic mice results in a neoplastic disease resembling mastocytosis, is a remarkable phenomenon, and best explained by the ‘multi-hit’ theory of cancer development [70-72]. Therefore, although the disease is clearly an artifical system, it may be useful and informative as an experimental model to study the develpment of MC tumors. First, different types of MC tumors were found in these mice, including indolent forms and more aggressive disease variants, similar to the situation in human SM. Second, neoplastic MC in mouse tissues exhibited morphological properties and phenotypic properties of neoplastic human MC [74]. Third, like in humans, mastocytosis was often accompanied by an associated hematologic non-MC-lineage disease/neoplasm (AHNMD) as has been reported for human SM [7, 8]. These associated tumors, however, were mostly of lymphoid origin as opposed to human mastocytosis (SM-AHNMD where mostly myeloid neoplasms are found), which is best explained by the lymphoma-specific trigger (NPM-ALK) used to induce the MC disease in mice. In this regard, it is also noteworthy that neoplastic MC in human mastocytosis did not express ALK. On the other hand several of the IL-9/NPM-ALK+ mice developed plasmocytomas, a lymphoid neoplasm that is a relatively frequent AHNMD in human SM. Moreover, sometimes, Non Hodgkin's lympohomas may be detectable in SM, and in these patients, lymphoma cells may express IL-9 receptors (H.H. and P.V., unpublished observation). Finally, neoplastic MC but not normal MC express several lymphoid marker antigens, such as CD2 and CD25 as well as CD30 [68, 69, 75]. With regard to CD25 and CD30, these data were confirmed in the present study. A remarkable aspect is that CD30 is primarily detectable in advanced SM [69, 75], and that this antigen (Ki-1) is otherwise also expressed in NPM/ALK+ lymphomas. Whether expression of CD30 is associated with overexpression of NPM in MC in advanced SM remains unknown. Alternatively, abnormal expression of both antigens is regulated by the same mechanisms in neplastic MC.

It is well established that murine MC express receptors for IL-9 and are responsive to this cytokine [27]. Therefore, we asked whether NPM/ALK/IL-9-dependent MC and neoplastic human MC (HMC-1 cells) display IL-9 receptors. In these experiments we found that HMC-1 cells express detectable IL-9 receptor transcripts, and LPS was found to augment IL-9 receptor expression. In addition, we were able to show that HMC-1 cells display surface IL-9 receptors by flow cytometry. Finally, we were able to show that primary bone marrow MC in patients with SM react with an antibody against the IL-9 receptor, and as mentioned above, the human MC leukemia cell line HMC-1 also expressed the IL-9 receptor. Supporting these observations, we also found that IL-9 promotes the growth HMC-1 cells *in vitro*. All these observations suggest that IL-9 might be an important cytokine contributing to the pathogenesis of SM, and that IL-9 may act as an autocrine or paracrine growth regulator. Notably, we found that HMC-1 cells and primary MC not only exhibit IL-9 receptors but also IL-9. In addition, IL-9 may be provided by surrounding cells in the tissues (e.g. by eosinophils or by TH2-cells), thereby promoting MC development in MC hyperplasia and SM. All in all, IL-9 and IL-9 receptors may indeed play a hitherto unrecognized role in the pathogenesis of SM, an observation that is in line with the notion that IL-9 is a growth regulator for normal MC [27].

The MC-transforming potential of the NPM-ALK kinase is of particular interest. MC usually grow under the influence of SCF, the natural ligand of the KIT tyrosine kinase receptor [9-13]. However, other tyrosine kinase receptors are also able to trigger MC growth or may even substitute for KIT in *KIT*-deficient animals. Likewise, it has been described that ErbB can substitute for KIT as kinase to promote MC development in *Kit*-deficient mice [50]. In addition, ErbB expression may even lead to the development of an MC neoplasm in mice [49]. Together with our data, these observations suggest that various tyrosine kinases (not only KIT) can trigger MC growth and survival, and can facilitate the development of MC tumors in mice. To which extent tyrosine kinases other than KIT play a role in naturally occurring (spontaneous) MC tumors in mice, remains at present unknown.

The morphology and phenotpye of neoplastic MC in IL-9/NPM-ALK+ mice was of interest since many features were found to resemble neoplastic MC in humans [74]. Likewise, MC were found to be either immature or mature cells, depending on the type of the MC disease and aggressiveness of the neoplasm, similar to human SM [74]. In addition, neoplastic MC were occasionally found to be spindle-shaped MC similar to human neoplastic MC [1-8, 74]. A remarkable finding was that neoplastic MC displayed CD25, a mastocytosis-related marker that is selectively expressed on neoplastic MC, but not in normal tissue MC in humans [75, 76]. In addition, neoplastic murine MC were found to express KIT and tryptase as well as CAE corresponding to human (neoplastic) MC [77, 78]. In summary, several different morphologic and phenotypic properties of MC in IL-9/NPM-ALK+ mice are highly reminicent of mastocytosis. On the other hand several questions concerning the impact/role of IL-9 and NPM or ALK in human SM, a disease that is considered to develop on the basis of a mutated KIT receptor (mostly KIT D816V), remain open. Likewise, it remains unknown whether KIT D816V or the murine equivalent KIT D814V would cooperate with IL-9 or NPM (or ALK) in inducing SM or triggering the progression of SM. In this regard it is noteworthy that neoplastic SM in advanced (human) SM were found to display substantial amounts of NPM, and sometimes even cytoplasmic NPM was detectable in these MC. Again, however, the possible cooperation of NPM and KIT or other factors (cytokines like IL-9) in the evolution of SM remains at present unknown. Here, novel robust mouse models including mice transgenic for murine or human *KIT* [79, 80], when combined with IL-9+ or NPM+ animals, should provide answers to these questions in future studies.

An interesting aspect in the current study was the simultaneous development of MC neoplasms and lymphomas in individual animals. Both neoplasms apparently were derived from immature lymphohematopoietic progenitor (stem) cells and both were found to express ALK. In this regard it is noteworthy that MC in human SM are also considered to derive from immature (multipotent) hematopoietic progenitors [7, 8, 81]. It was therefore of particular importance to delineate both diseases by several different approaches. In a first step, we were able to show that both neoplasms expressed different phenotypes. In fact, neoplastic MC did not express any of the T cell- or B cell-restricted antigens analyzed (except ALK), and the T cell- and B cell-lymphomas did not express MC antigens such as KIT, tryptase, or chymase. In a second step, we asked whether tumor-specific lymphoma stem cells in established ALK+ lymphomas can give rise to MC neoplasms in secondary recipients. However, although secondary recipients were found to develop ALK+ lymphomas in most cases, no MC neoplasms could be detected in any of these animals independent of the presence or absence of an IL-9-background. These data support the conclusion that NPM-ALK-transformed cells can be separated into two different categories, namely MC-committed (MC-neoplasm-committed) progenitors and lymphoma-committed (B cell- or T cell-committed) progenitors. In addition, some of these progenitors may be extremely immature without differentiation-capacity – which then would result in the formation of extremely immature (undifferentiated) myelosarcoma-like tumors that were also found to develop in some of the mice in this study.

In summary, our data show that IL-9 and NPM-ALK cooperate in producing a mastocytosis-like disease in mice that resembles a primary MC disease in several different aspects, and may thus be a useful model for studying the development and pathogensis of mastocytosis.

## Abbreviations used

ALK: Anaplastic lymphoma kinase
BM: Bone marrow
CAE: Naphthol AS-D chloroacetate esterase
FCS: Fetal calf serum
IL: Interleukin
IL-9: Interleukin-9
MC: Mast cell(s)
NPM: Nucleophosmin
Pc/pb: Plasmocytic/plasmoblastic
SCF: Stem cell factor
SM: Systemic mastocytosis
rm: Recombinant mouse
